# New observations of fluorescent organisms in the Banda Sea and in the Red Sea

**DOI:** 10.1371/journal.pone.0292476

**Published:** 2024-06-12

**Authors:** Lars Henrik Poding, Peter Jägers, Budiono Senen, Gino Valentino Limmon, Stefan Herlitze, Mareike Huhn

**Affiliations:** 1 Department of General Zoology and Neurobiology, Institute of Biology and Biotechnology, Ruhr-University Bochum, Bochum, Germany; 2 Universitas Banda Neira, Maluku Tengah, Indonesia; 3 Fisheries and Marine Science Faculty, Pattimura University, Ambon, Indonesia; 4 Maritime and Marine Science Center of Excellence, Pattimura University, Ambon, Indonesia; 5 Center for Collaborative Research on Aquatic Ecosystem in Eastern Indonesia, Ambon, Indonesia; King Abdulaziz University, SAUDI ARABIA

## Abstract

Fluorescence is a widespread phenomenon found in animals, bacteria, fungi, and plants. In marine environments fluorescence has been proposed to play a role in physiological and behavioral responses. Many fluorescent proteins and other molecules have been described in jellyfish, corals, and fish. Here we describe fluorescence in marine species, which we observed and photographed during night dives in the Banda Sea, Indonesia, and in the Red Sea, Egypt. Among various phyla we found fluorescence in sponges, molluscs, tunicates, and fish. Our study extends the knowledge on how many different organisms fluoresce in marine environments. We describe the occurrence of fluorescence in 27 species, in which fluorescence has not been described yet in peer-reviewed literature. It especially extends the knowledge beyond Scleractinia, the so far best described taxon regarding diversity in fluorescent proteins.

## Introduction

Fluorescence is the property of a molecule to absorb light of a certain wavelength followed by the emission of light with longer wavelength. In bacteria, plants, fungi, and animals, various fluorescent molecules have been described such as chitin, minerals, carotenoids, flavonoids, porphyrins, chlorophyll, phycobiliproteins or green fluorescent protein (GFP) and (GFP)-like fluorescent proteins [1–3].

In marine environments many fluorescent species have been described and for some of these species an ecological, behavioral and/or physiological function has been suggested [2–4]. In the jellyfish *Aequorea victoria*, for example, where the first fluorescent protein (avGFP) was characterized, GFP is expressed as an acceptor molecule for bioluminescent light [5]. So far fluorescent proteins (FP) have been found in Cnidaria, Vertebrata, Cephalochordata (lancelets), and Arthropoda, but have not been described (i.e. published in peer-reviewed literature) in Porifera (sponges), Ctenophora (comb jellies), Tunicata (tunicates), Hemichordata (hemichordates), Echinodermata (echinoderms), Mollusca (molluscs), Annelida (segmented worms), and Nematoda (roundworms) [2,6–11]. Most fluorescent proteins with blue to red emission spectra (GFP-like fluorescent proteins) have been identified in Anthozoa (e.g. corals and anemones) [6]. There, the fluorescence has been hypothesized to be involved in attraction of photosynthetic symbionts, in photoprotection of the symbionts and in converting blue light to longer wavelength light in mesophotic environments (photoenhancement) [2,4] where the green to red components of the sunlight are selectively removed with water depth resulting in a blue light environment [2,4]. In fish, red fluorescent body coloration and red fluorescent iris have been proposed to be involved in intraspecific communication [12–16]. In addition, differences in the fluorescence pattern between males and females have been suggested to play a role in sexual communication [13,17].

In contrast to jellyfish, corals, anemones, copepods, lancelets, eels, and moray eels, where fluorescence is based on the expression of fluorescent proteins, non-FP based fluorescence has been observed in Porifera (sponges), Heterobranchia (slugs and snails), Annelida (segmented worms), Crustacea (crustaceans), Osteichthyes (bony fish), and Chondrichthyes (cartilaginous fishes) [8,18–22].

Here we show photographs of new cases of fluorescence in marine species, which were taken during night dives in the Banda Sea, Banda Islands, Indonesia and the Red Sea, Dahab, Egypt. These fluorescent species include nudibranchs, tunicates, sponges, various other invertebrates, and fish. In addition to these new records of fluorescence in marine species, we show detailed Leica THUNDER microscopy images of known fluorescent species to provide precise information about the distribution of fluorescence in different body parts.

## Methods

### Ethics statement

No permits were required for the in-situ images as specimen were neither collected nor handled, and photos were taken during recreational Scuba dives. For the THUNDER images, fish and invertebrates were purchased from the wholesale trader DeJong Marinelife (Netherlands) in 2021/2022 or from Korallenfarm Joe & Co (Germany).

### Photo acquisition in the field

Images of fluorescent species were acquired during SCUBA diving in the Red Sea in Dahab, Egypt (28°29’20.0”N 34°30’57.2”E) and in the Banda Sea at the Banda Islands, Maluku, Indonesia (4°30’55.62”S 129°53’35.92”E). All dives were started after sunset and limited to a maximum depth of 15 meters, which was monitored by a dive computer. Field observations were carried out in May 2019 (Red Sea) and in February-March 2019 and September 2022 (Banda Sea).

**Fig 1:**
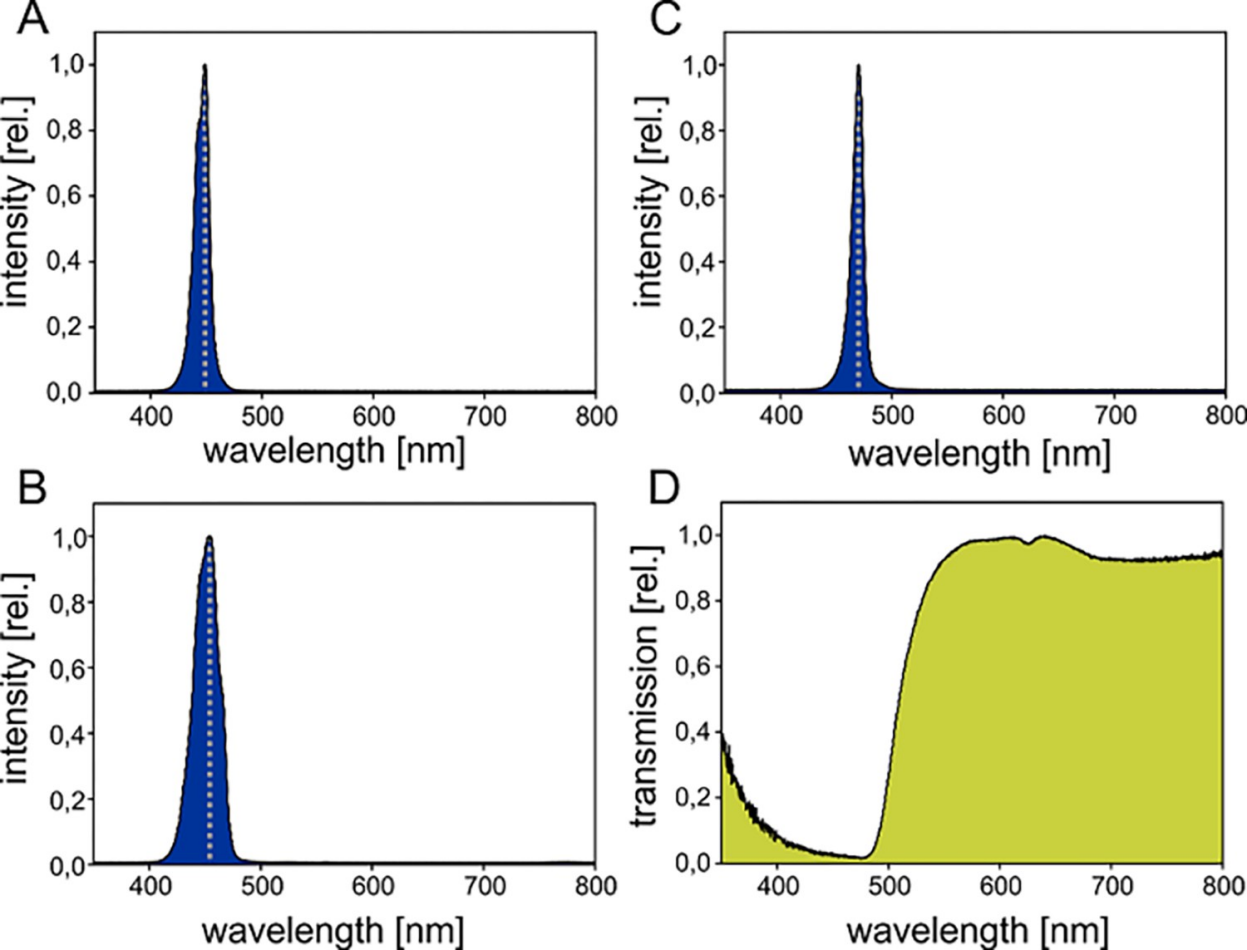
Fluorescence was excited with “Sola” light source (NightSea, USA; ʎ _max_ = 449 nm; A), Flashlight (D2000; INON; Japan) with Ex-Inon excitation filter (NightSea, USA; ʎ _max_ = 454 nm; B) or “Blue Star” light source (NightSea, USA; ʎ _max_ = 470 nm; C). Transmission of the yellow plastic filter BF 2 (NightSea, USA; E) showing a low cut at ʎ < 500 nm. Relative intensity (A-C) and transmission (D) were determined with a Spectrometer (Flame S-UV-VIS-ES, Ocean Optics, USA). A halogen Light Source (HL-3 plus, Ocean Optics, USA) was used to measure transmission of yellow long pass filter.

Fluorescence was excited with “Sola” light source (NightSea, USA; ʎ max = 449 nm), Flashlight (D2000; INON; Japan), Ex-Inon excitation filter (NightSea, USA; ʎ max = 454 nm) (Fig 1) or “Blue Star” light source (NightSea, USA; ʎ max = 470 nm). The plastic transmission filter (BF 2, NightSea, USA) in front of the underwater housing showed a low cut at ʎ < 500 nm (Fig 1). Fluorescence photography was performed with Powershot G15 (Canon, Japan) placed in a WP DC 48 (Canon, Japan) underwater housing or Sony α 6000 (Sony, Japan) placed in a SF-A6500 (Seafrogs, Hongkong) underwater housing (details on emission and transmission spectra of light sources and filter specified in Fig 1, details on camera specifications and settings in S1 File). All images were processed with Lightroom (Version 2015.1.1, Adobe, USA) and Corel Draw (Version 20.0.0.633, Corel Corporation, Canada), by correcting the brightness and contrast uniformly throughout the entire image and cropping the images (example of digital processing provided in S1 File).

Species were identified with standard literature [23–32]. The species were identified by comparison of digital underwater pictures with the description and pictures in the literature.

### Photo acquisition by Leica THUNDER microscopy

Detailed images of *Anampses meleagrides*, *Cirrhilabrus aquamarines*, *Cirrhilabrus rubrisquamis*, *Doryrhamphus excisus*, *Eviota atriventris*, *Eviota nigriventris*, *Lybia tessellata*, *Nembrotha kubaryana*, *Odontodactylus scyllarus*, *Synchiropus sycorax*, and one species of Polyplacophora were acquired with the fluorescence THUNDER stereo microscope Leica M205 FCA equipped with a DFC90000 GT Camera and a GFP (ʎ _Ex_: 395–455 nm, ʎ _Em_: 480 long pass filter), mCherry (ʎ _Ex_: 540–580 nm, ʎ _Em_: 593–667 nm), and a CY5 (ʎ _Ex_: 590–650 nm, ʎ _Em_: 663–737 nm) filter cube. All images were processed with LASX (Version 3.7.6.25997, Leica, Germay), Lightroom (Version 2015.1.1, Adobe, USA), and Corel Draw (Version 20.0.0.633, Corel Corporation, Canada) by correcting the brightness and contrast uniformly, and cropping the images.

## Results

We could identify fluorescence in 27 marine species that had not been described to be fluorescent so far. The species in which the new fluorescent signals were observed belonged to the phyla Porifera, Mollusca, Arthropoda, Annelida, and Chordata.

**Fig 2:**
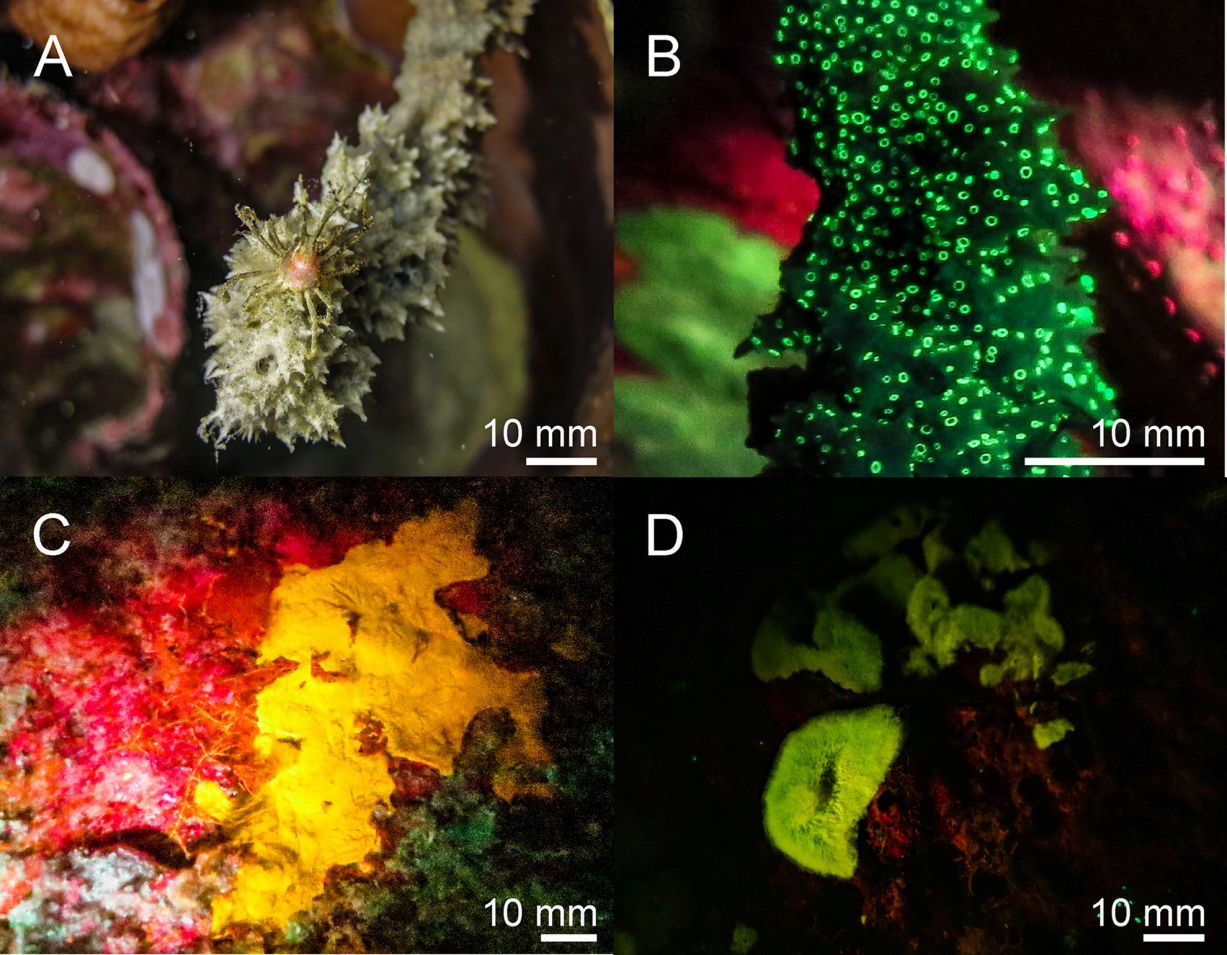
Fluorescence in Porifera in the Banda Sea: The thorny stem sponge *Gelliodes fibulata* ((A & B) white light in A, fluorescence in B) shows green fluorescent spots. Two unidentified sponges (C, D) fluoresce yellow and green. The scale bars are approximated based on published sizes of the respective organism.

Porifera–We found green fluorescent spots on the surface of the sponge *Gelliodes fibulata* (Fig 2A and 2B). Furthermore, we observed yellow and orange fluorescence in different unidentified sponges in the Red Sea (Fig 2C) and Banda Sea (Fig 2D).

**Fig 3:**
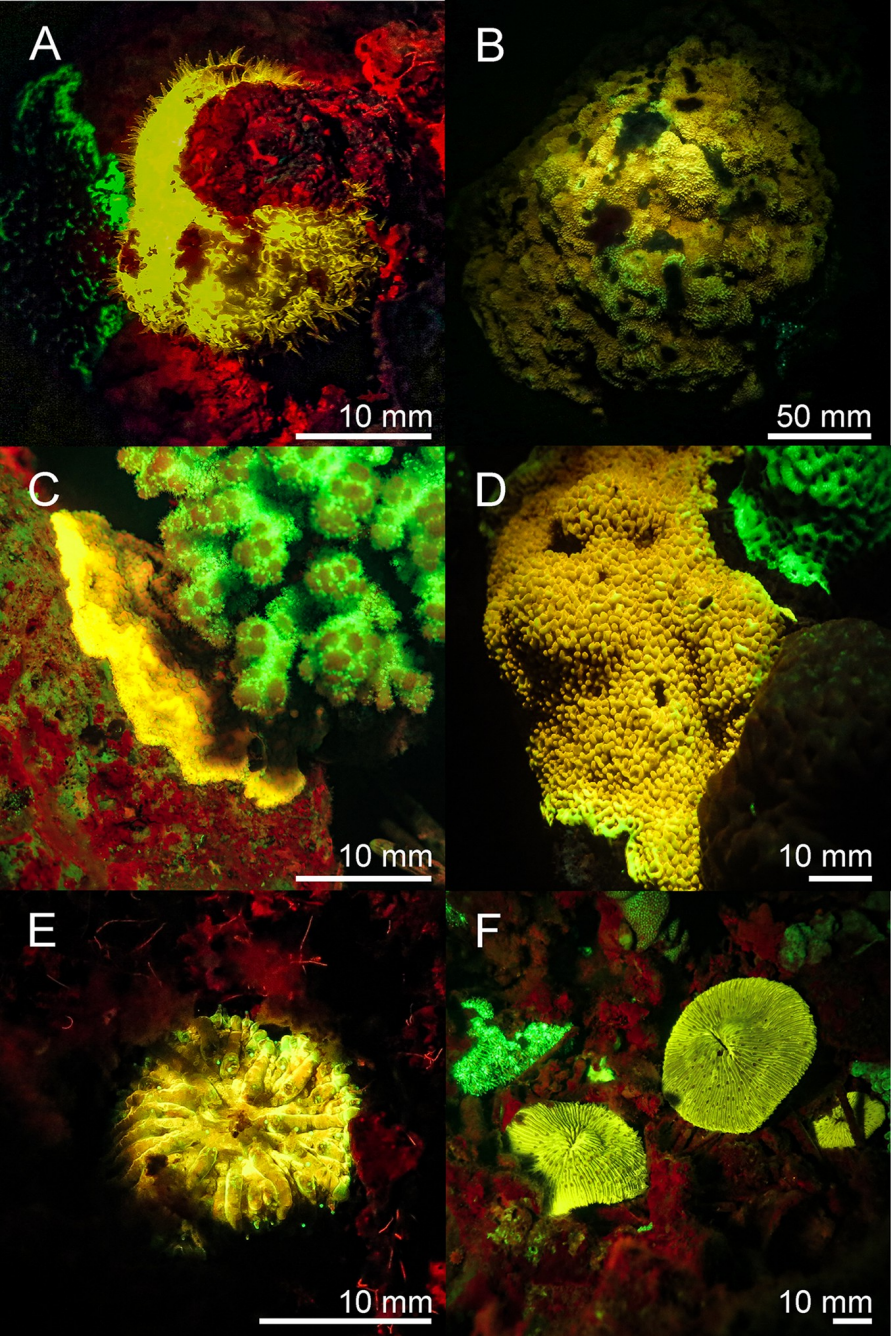
Fluorescence in corals: Yellow fluorescent corals in the Banda Sea and in the Red Sea. *Physogyra lichensteinii* (A), *Montipora sp*. (B—D), and *Cycloseris* sp./*Fungia sp*. (E & F). A, B, D–F: Banda Sea, C: Red Sea. The scale bars are approximated based on published sizes of the respective organism.

Scleractinia–We found six species of stony corals that exhibited yellow fluorescence (Fig 3), *Physogyra lichtensteini*, three different species of *Montipora*, and two *Cycloseris/Fungia* species.

**Fig 4:**
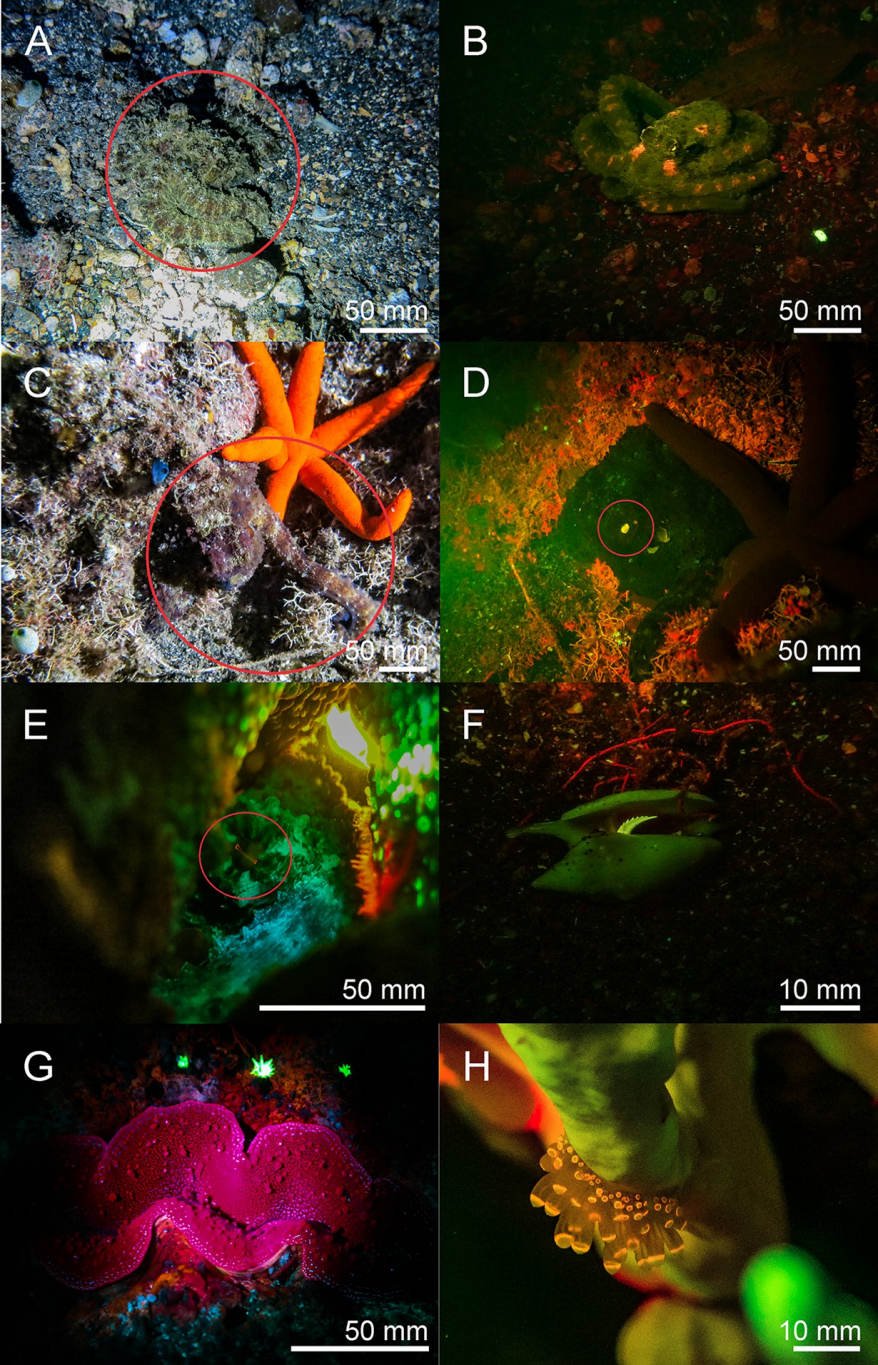
Fluorescence in Mollusca: Three different types of fluorescent Octopods; *Abdopus aculeatus* (A (white light) & B (fluorescence)) with orange fluorescent skin patches, two unidentified species of Octopodidae (C (white light)–E (fluorescence)) with fluorescent eyes, green fluorescent operculum of *Euprotomus bulla* (F), red fluorescent mantle of *Tridacna crocea* (G), orange fluorescent *Facelina rhodopus* (H) on *Millepora sp*. A–G: Banda Sea, H: Red Sea. The scale bars are approximated based on published sizes of the respective organism.

**Fig 5:**
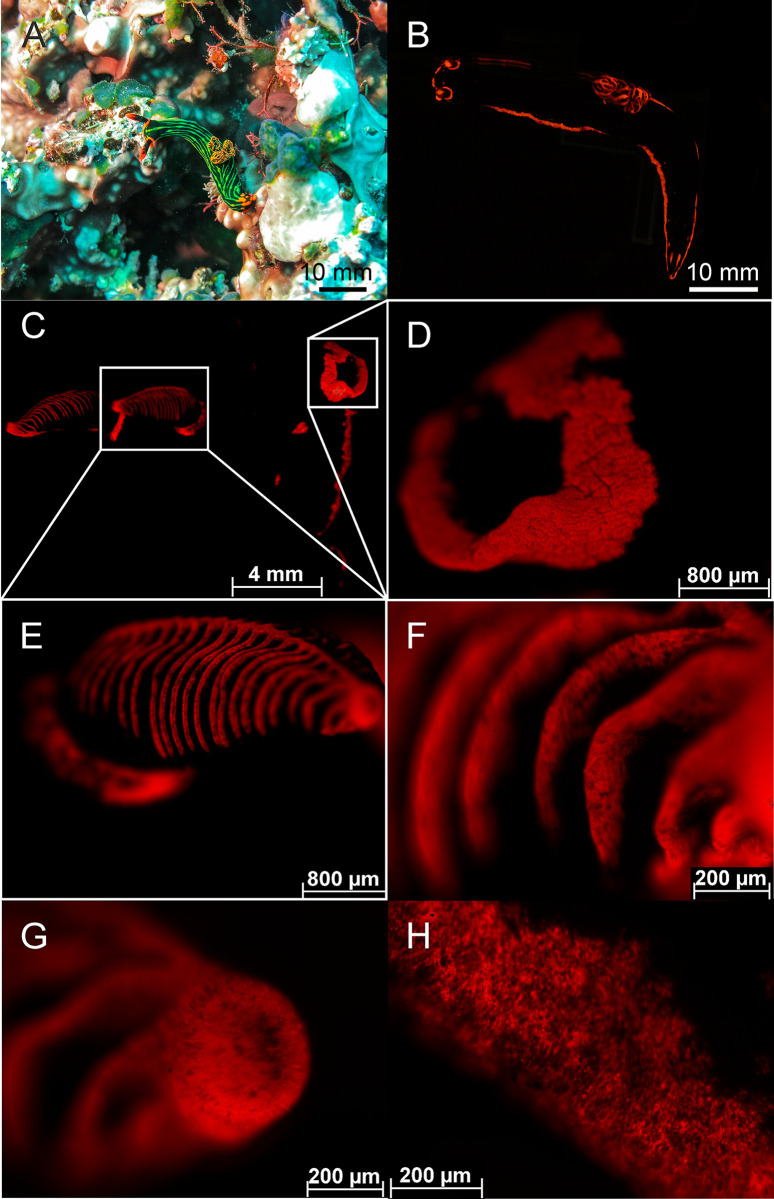
Fluorescence in the nudibranch *Nebrotha kubaryana*: (A) natural habitat (Banda Sea), white light & Fluorescence (B), whole body (B, C), oral tentacles (D), rhinophores (E-G), gill (H) fluorescence. Pictures C–H created with a THUNDER microscope (Leica, Germany) with CY5 filter. The scale bars (A & B) are approximated based on published sizes of the respective organism.

**Fig 6:**
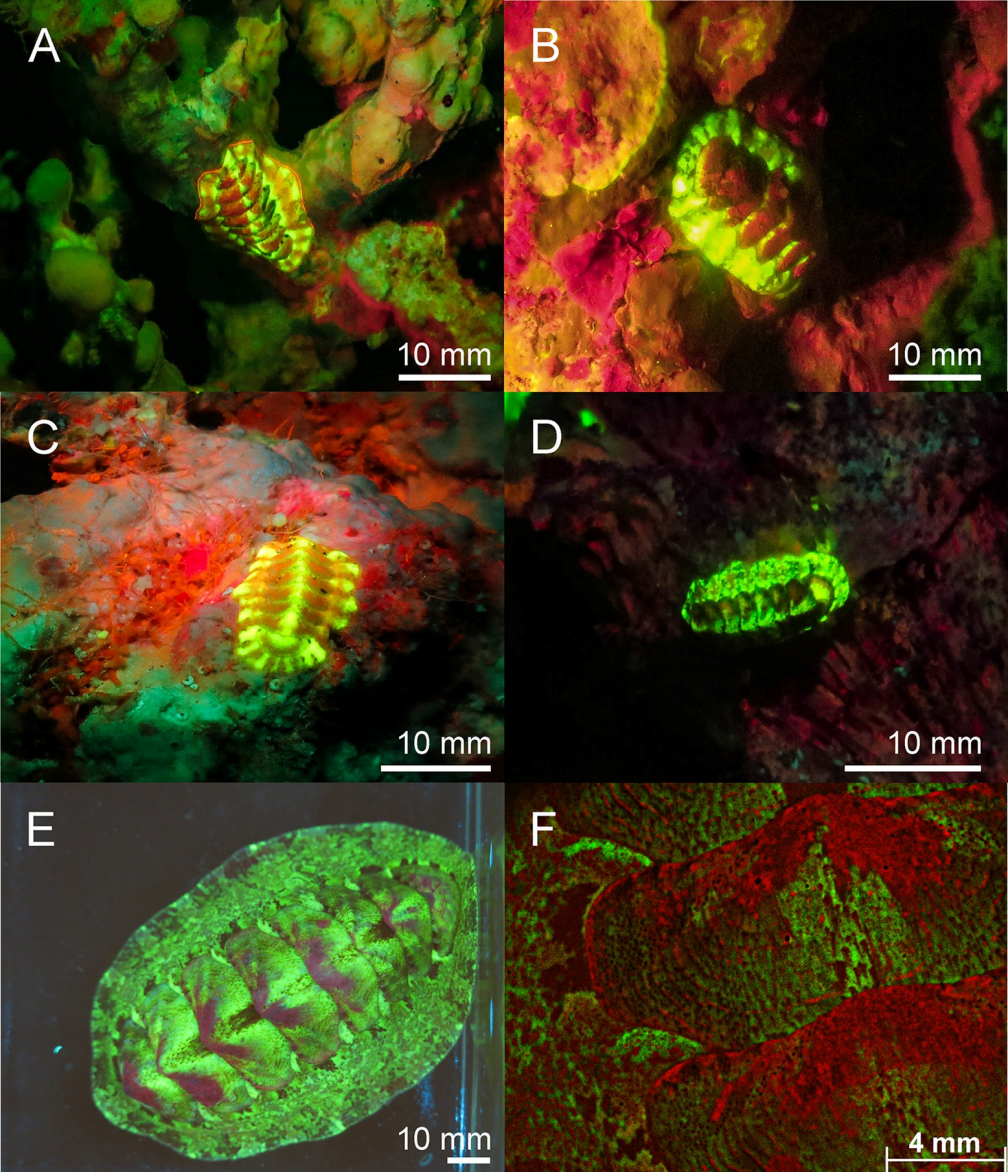
Fluorescence in Polyplacophora: Red, yellow and green fluorescent Polyplacophora in the Red Sea (A-C) and in the Banda Sea (D). Bright green and red fluorescent Polyplacophora in a reef tank in E-F. Picture F is created with a THUNDER microscope (Leica, Germany) with GFP & CY5 filter. The scale bars of A-E are approximated based on published sizes of the respective organism.

Molluscs—In molluscs we found fluorescence in the families Octopodidae, Strombidae, Cardiidae, Facelinidae (Fig 4), Polyceridae (Fig 5) and unidentified families of Polyplacophora (Fig 6). The octopus *Abdopus aculeatus* revealed orange fluorescent markings on its body. Bright fluorescent patches were located below the yellow fluorescent eyes and on every arm (Fig 4A & 4B). Other species (unspecified) of the same family showed no fluorescent body markings, but had yellow fluorescent eyes (Fig 4B, 4D and 4E). We found green and red fluorescence in several snails. The bubble conch *Euprotomus bulla* had a bright green fluorescent operculum and weak green fluorescent eyes. The giant clam *Tridacna crocea* showed bright red fluorescence (Fig 4G). In addition, the nudibranch *Facelina rhodopos* (Fig 4H) showed bright orange fluorescence at the tip of the cerata. Another nudibranch *Nembrotha kubaryana* that we photographed in the Banda Sea in its natural habitat (Fig 5A) and had purchased from DeJong Marinelife (Netherlands) to image with THUNDER microscopy (Fig 5B) revealed strong red fluorescence. We were able to localize the fluorescence in more detail under laboratory settings and found fluorescent patches around the mouth (Fig 5D), the rhinophores (Fig 5E–5G), gills (Fig 5B), and the dorsal rim (Fig 5B & 5H). We also found a variety of different Polyplacophora that exhibited bright green, yellow, and red fluorescence (Fig 6A–6D).

**Fig 7:**
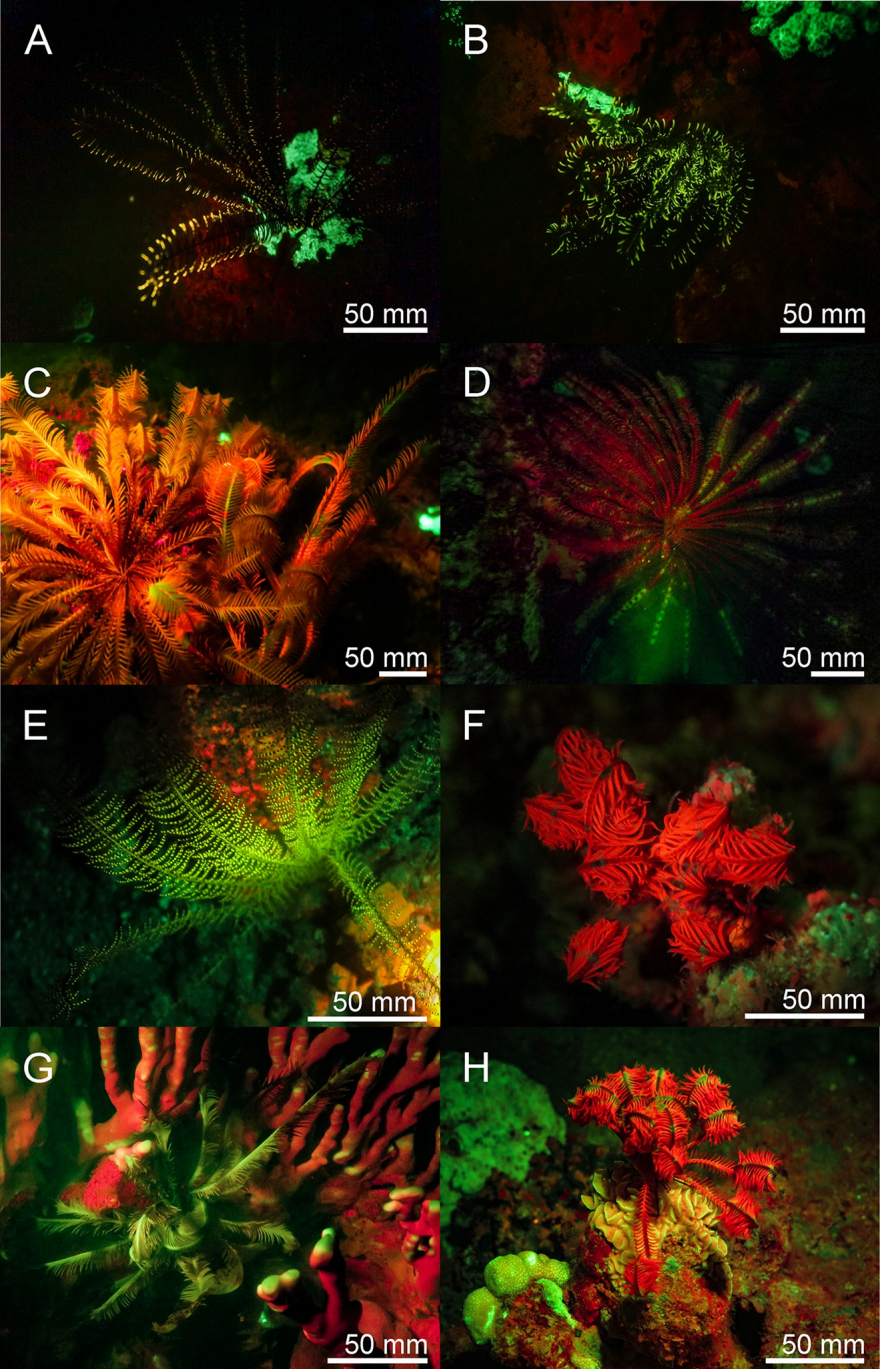
Fluorescence in echinoderms: New observations of red, yellow and green fluorescence in crinoids from the Banda Sea (A-E) and in the Red Sea (F-H). The scale bars are approximated based on published sizes of the respective organism.

Echinodermata–We found bright green, yellow, and red fluorescent crinoid species of different genera in the Banda Sea and in the Red Sea (Fig 7).

**Fig 8:**
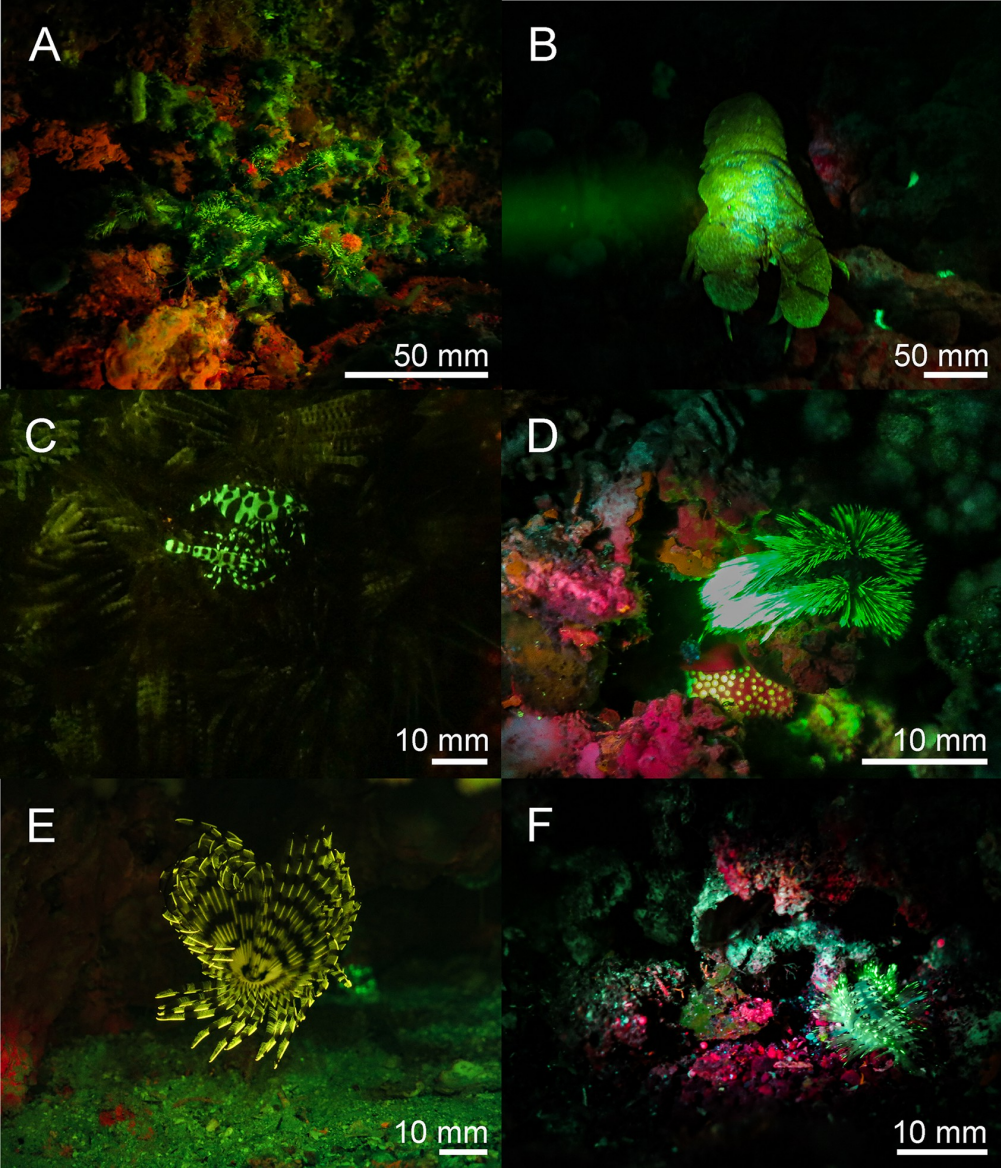
Fluorescence in Arthropoda and Annelida: Green fluorescent *Camposcia retusa* (A), *Scyllarides sp*. *(B)*, *Periclimenes colemani* (C), Eunicida (D, F), Yellow fluorescent chaeta of an unidentified species of Sabellidae (E). A, C, D, F: Banda Sea, B & E: Red Sea. The scale bars are approximated based on published sizes of the respective organism.

**Fig 9:**
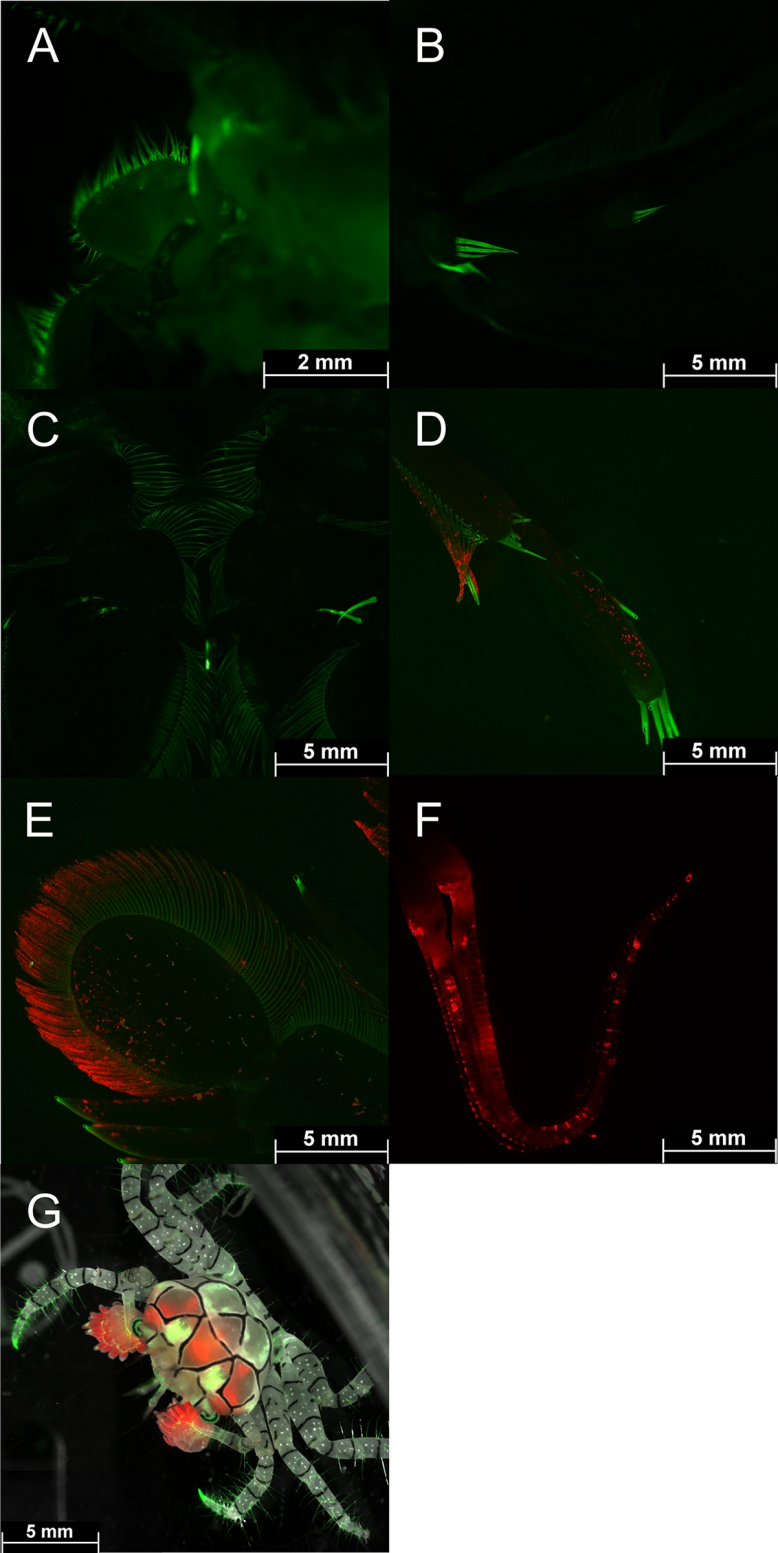
Fluorescence of *Odontodactylus scyllarus* and *Lybia tessalata*: Green (GFP filter) and red fluorescing (Cy5 filter) structures of maxillipeds (A, B, F), pleopods(C), uropods (E), and paraeopods (D). Green and red fluorescing *Lybia tessellata* (G; overlay of brightfield, GFP & CY5 filter). Pictures were taken with a THUNDER microscope (Leica, Germany).

Arthropoda—Several fluorescent Decapoda of the family Inachidae, Scyllaridae and, Palaemonidae were found. The decorator crab *Camposcia retusa* (Fig 8A Banda Sea) showed a bright green fluorescent carapace. In the Red Sea we discovered a bright green fluorescing slipper lobster *Scyllarides sp*. (Fig 8B). In addition, we found bright green fluorescence in the carapace of Coleman`s Shrimp, *Periclimenes colemani* (Fig 8C), living in symbiosis with fire urchins that do not fluoresce. Green and red fluorescence of the carapace was discovered in peacock mantis shrimps *Odontodactylus scyllarus*. The THUNDER images revealed fluorescence in filamentous structures on maxillipeds (Fig 9A, 9B and 9F), pleopods (Fig 9C), uropods (Fig 9E), and on paraeopods (Fig 9D) of *Odontodactylus scyllarus*. Furthermore, we identified fluorescent body parts of the mosaic boxer crab (*Lybia tessellata*) (Fig 9G).

Annelida—We also found fluorescence in two types of bristle worms (polychaetes) from the families Eunicidae and Sabellidae. In the subclass Eunicida only the bristles fluoresced bright green (Fig 8D and 8F). In addition, we found bright yellow fluorescence in a feather duster worm (Sabellidae) (Fig 8E).

**Fig 10:**
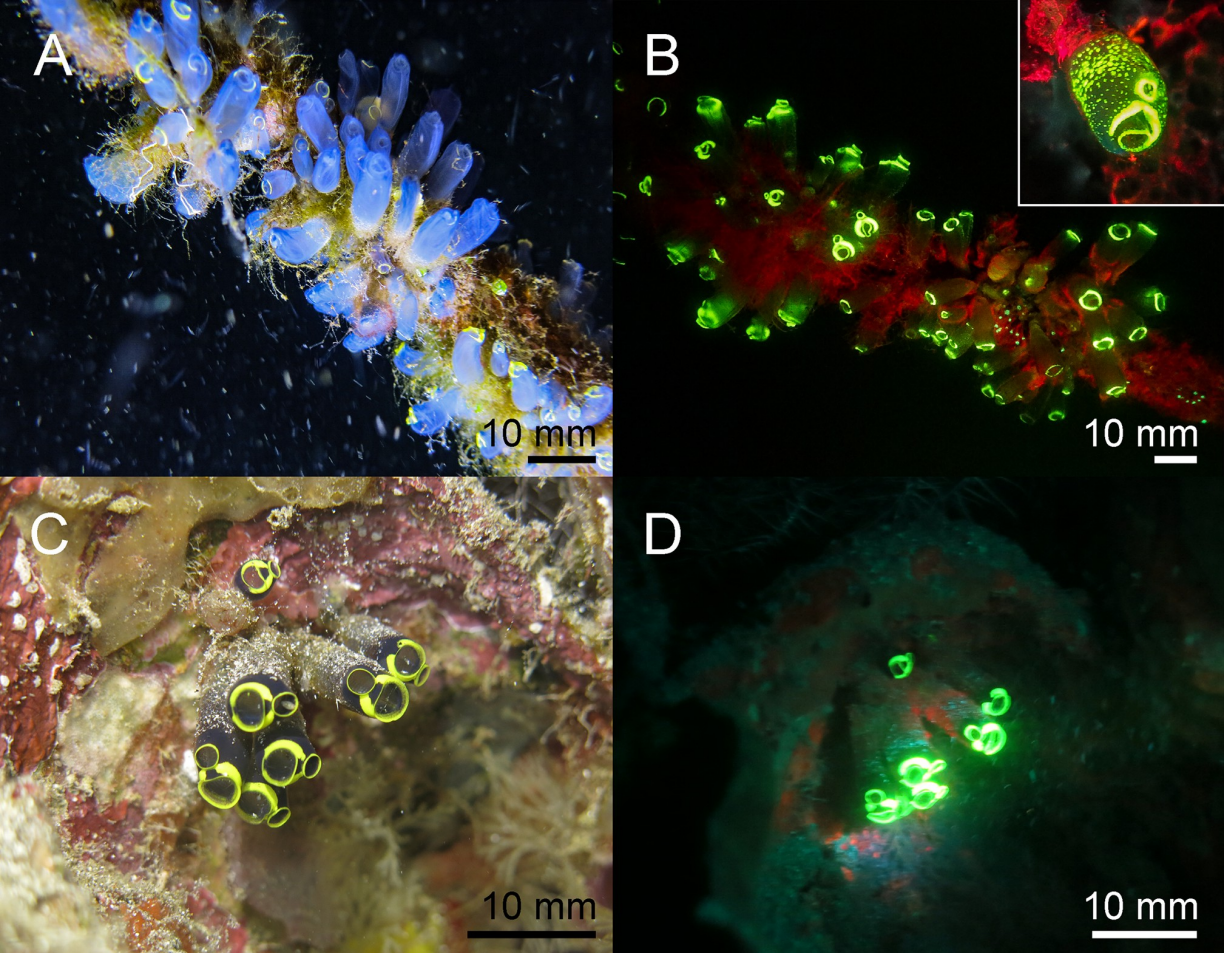
Fluorescence in ascidians in the Banda Sea: White light and fluorescent picture of *Clavelina coerulea* ((A, B) white light in A, fluorescence in B), *Clavelina robusta* ((C, D) white light in C, fluorescence in D). The green fluorescence accumulates on the siphons of the ascidians. The scale bars are approximated based on published sizes of the respective organism.

**Fig 11:**
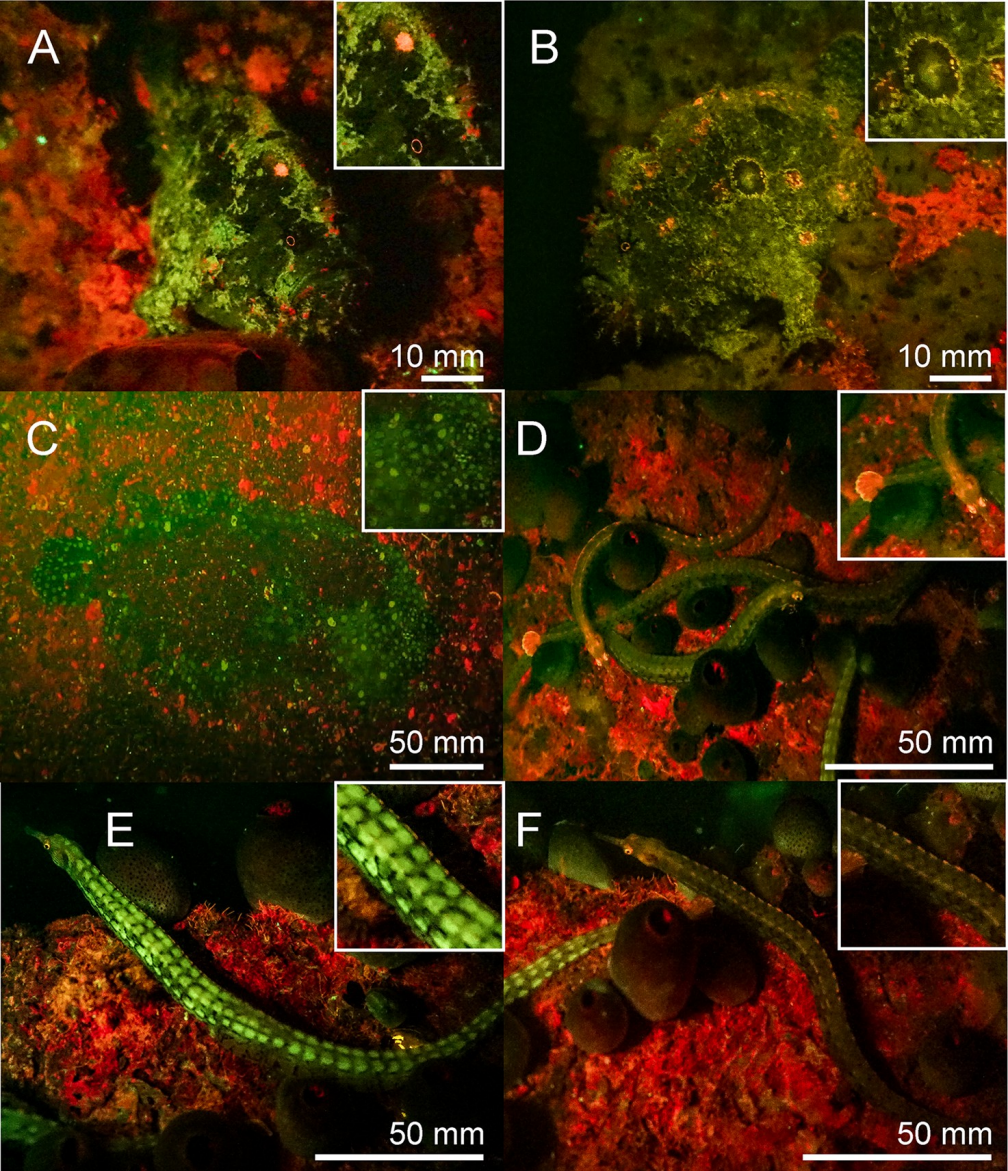
Fluorescence in fish in the Banda Sea: Green and orange fluorescing *Antennatus coccineus* (A, B), green fluorescing *Bothus pantherinus* (C) and different fluorescing individuals of *Corythoichthys intestinalis* (D-F). The scale bars are approximated based on published sizes of the respective organism.

**Fig 12:**
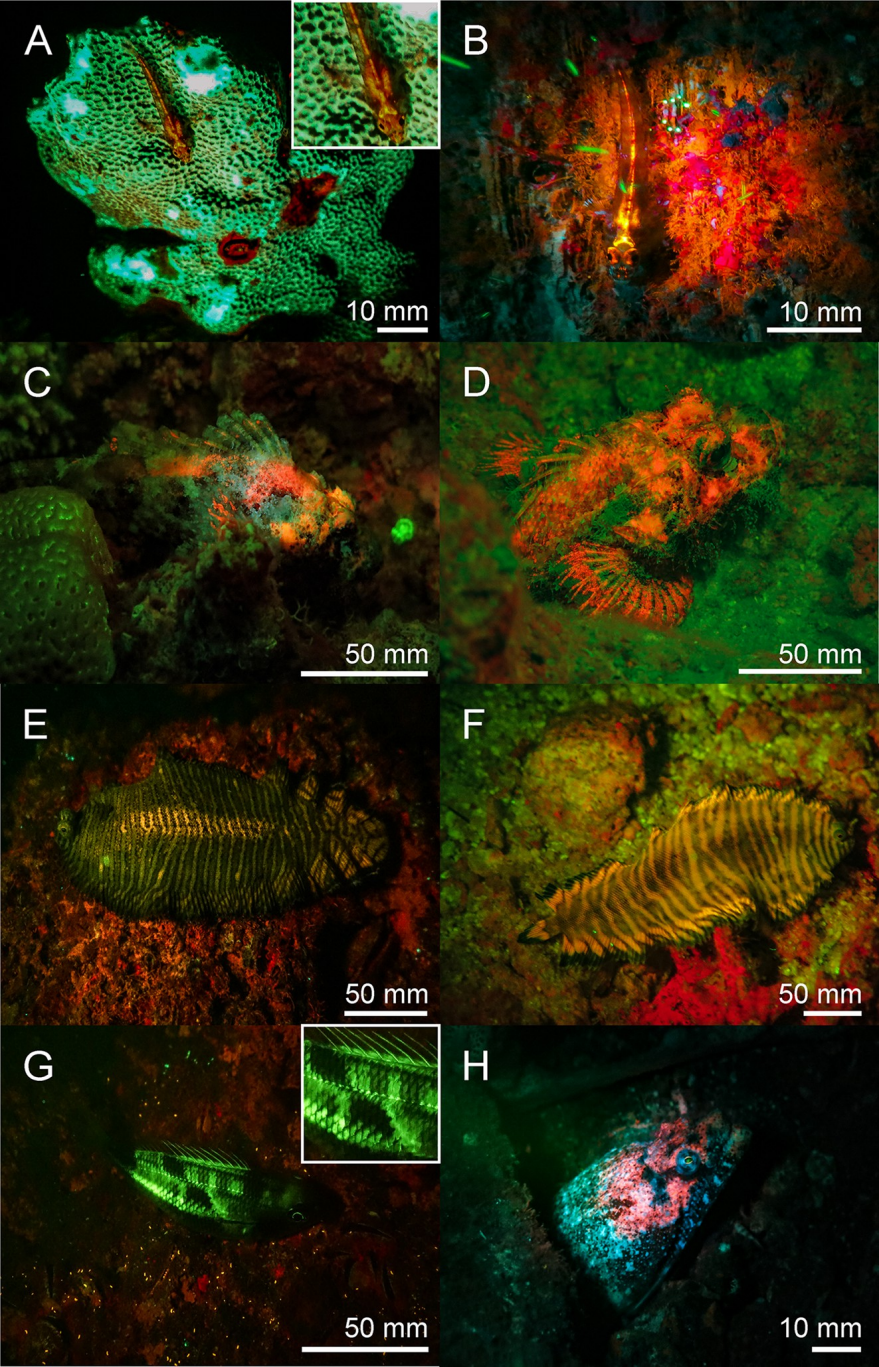
Fluorescence in fish: Red fluorescing *Pleurosicya mossambica* (A & B), *Scorpaenopsis possi* (C & D), different colormorphs of orange fluorescing *Soleichthys heterorhinos* (E & F), undefined species of Lutjanidae (G), and *Brachysomophis henshawi* (H) with a red fluorescing head. A, B, D, F-H: Banda Sea, C & E: Red Sea. The scale bars are approximated based on published sizes of the respective organism.

**Fig 13:**
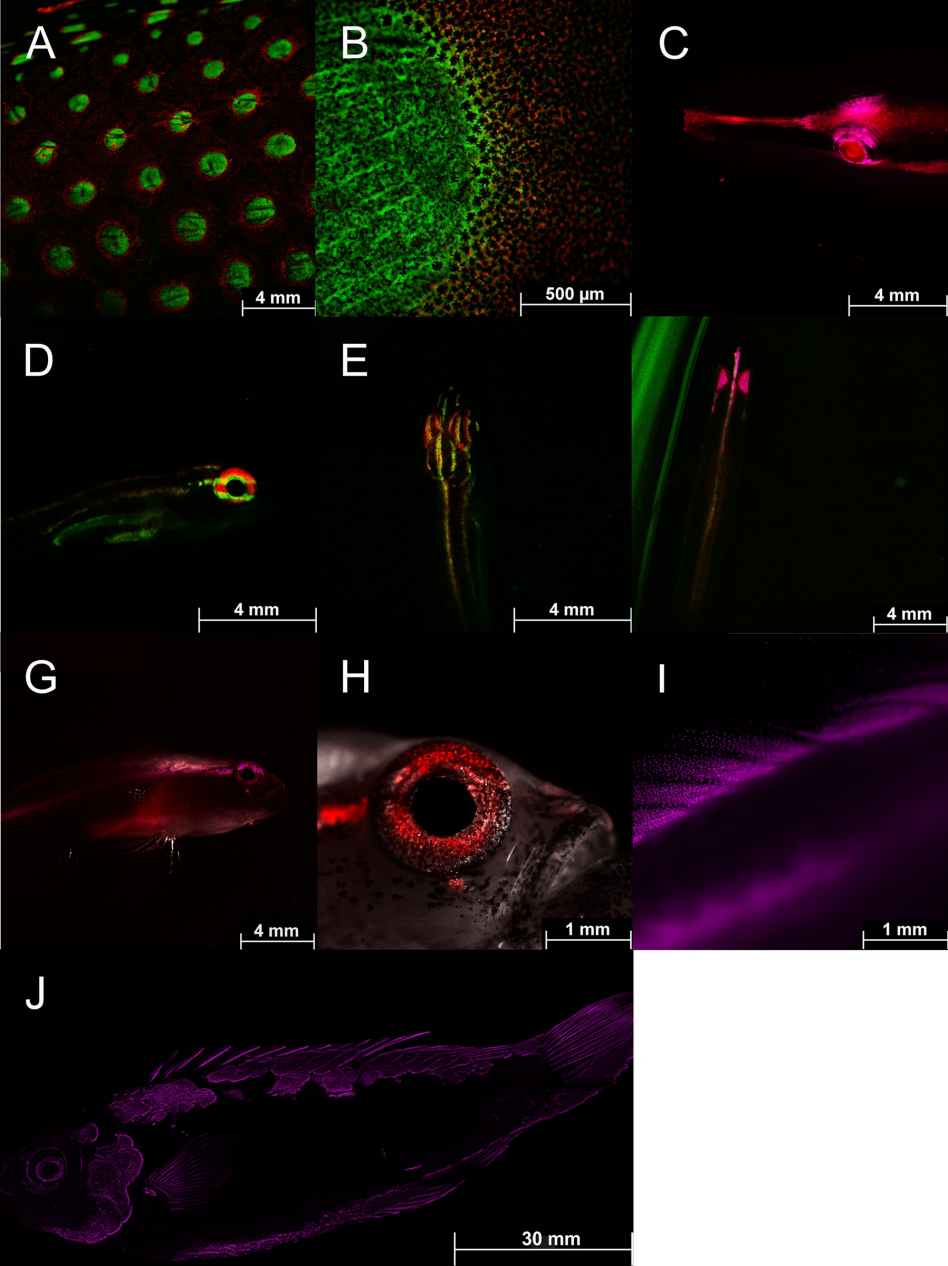
Fluorescence in fish: Detailed fluorescent pictures of *Anampses meleagrides* (A, B; both overlay of GFP and CY5 filter), *Doryrhamphus excisus* (C; overlay of mCherry and CY5 filter), *Eviota atriventris* (D, E; both overlay of GFP & CY5 filter), *Eviota nigriventris* (F-I; F: overlay of GFP, CY5, mCherry filter; G: overlay of mCherry and CY5 filter; H: overlay of brightfield & CY5 filter; I: mCherry filter), *Cirrhilabrus aquamarinus* (J; mCherry filter), Pictures were taken with a Thunder microscope (Leica, Germany).

**Fig 14:**
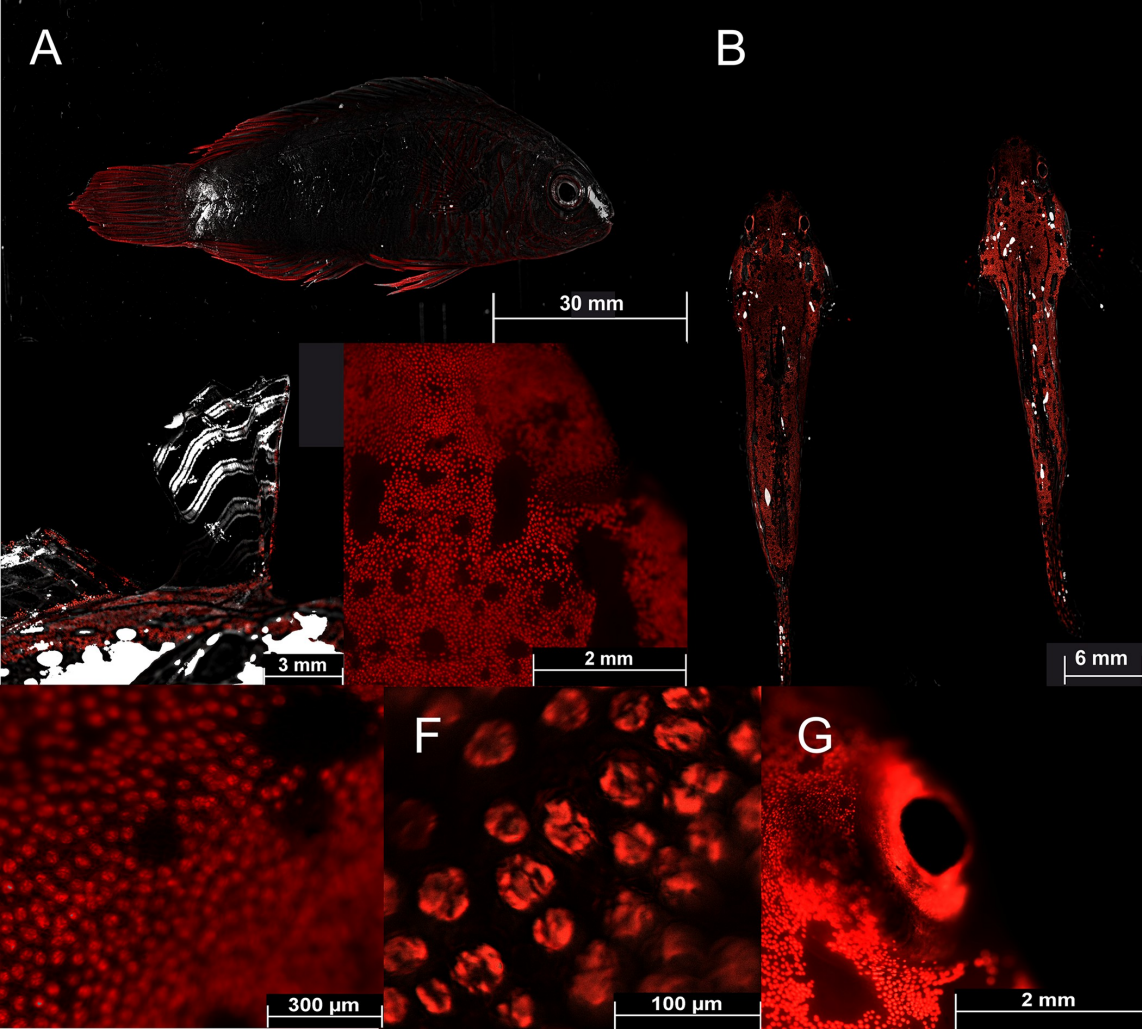
Fluorescence in fish: Detailed fluorescent pictures of *Cirrhilabrus aquamarinus* (A; brightfield & Cy5 filter), Pair of *Synchiropus sycorax* (B; ♀ left and ♂ right; overlay of brightfield and Cy5 filter), first dorsal fin of *Synchiropus sycorax* ♂ (C; overlay of brightfield and Cy5 filter), detailed fluorophores of *Synchiropus sycorax* (D-F; Cy5 filter), fluorescent patch around the eye of *Synchiropus sycorax* ♂ (G; Cy5 filter). Pictures were taken with a Thunder microscope (Leica, Germany).

Chordata–Two fluorescent ascidian species and three species of fluorescent bony fish were photographed in the Banda Sea. Both ascidians *Clavelina coerulea* (Fig 10A & 10B) and *Clavelina robusta* (Fig 10C & 10D) revealed a bright green rim-like fluorescence around their siphons. In both species the fluorescent rims appeared yellow under white light conditions. We found two green and orange fluorescent scarlet frogfish *Antennatus coccineus* (Fig 11A & 11B). The green fluorescence was distributed over the entire body, while the orange fluorescence was found in patches and in the lures. In addition, we found a bright fluorescing green leopard flounder *Bothus pantherinus* (Fig 11C). Green fluorescence was more prominent in the white banded sections of the skin. We also found individual differences in fluorescence in the banded pipefish *Corythoichthys intestinalis*. Individuals revealed either green or orange fluorescent skin patterns (Fig 11D–11F). However, all individuals showed a bright yellow/orange fluorescence around the eyes and the caudal fin (Fig 11D–11F). Moreover, we observed two individuals of *Pleurosicya mossambica* in the Banda Sea with bright orange-red fluorescence around the eyes and the spinal column (Fig 12A & 12B) and two individuals of *Scorpaenopsis possi* with orange and red fluorescent patches in their tissue (Fig 12C & 12D). In addition, we found two different color morphs of *Soleichthys heterorhinos* from the Banda Sea (E) and Red Sea (F) and a green fluorescent undefined species of Lutjanidae (G), and *Brachysomophis henshawi* with a red fluorescent head (H) (Figs 13 & 14).

## Discussion

During night dives in the Banda Sea, Indonesia and the Red Sea, Egypt we photographed marine species, in which, to our knowledge, fluorescence had not been documented in scientific journals before and for which fluorescence has not been characterized. These included species of sponges, crustaceans, polychaetes, slugs, snails, octopus, ascidians, and fish.

Various molecules and anatomical structures that mediate fluorescence have been identified in different species. These include fluorescent proteins, carotenoids, pteridine, porphyrins, tryptophane derivatives, and chlorophyll [2,22,33,34]. Fluorescent proteins have been found in a few taxa of metazoans including Cnidaria and Arthropoda, but also in Cephalochordata and Vertebrata [2,10]. Since we found fluorescence in two species of ascidians that are closer related to vertebrates than other invertebrates, next generation sequencing techniques can reveal if these ascidians contain FP-like genes. In both species bright green fluorescence was seen in a rim-like structure around the oral and atrial siphon (Fig 6). Since food is ingested via the oral siphon, fluorescence might be involved in attraction of plankton which has been demonstrated for the green fluorescence of the jellyfish *Olindias formosa* [35] and for green and orange fluorescence in corals [36].

In Arthropoda different molecules have been described to be fluorescent [9,37–39]. The blue fluorescence in the carapace of the lobster *Homarus gammarus* is mediated by a multimolecular carotenoprotein, α crustacyanin, binding to the carotenoid astaxanthin [40]. The cuticle of scorpions contains the alkaloid β-carboline which is derived from tryptamine and is excited by UV light leading to the emission of blue fluorescent light [41]. The *Drosophila melanogaster* mutant ‘sepia’ is characterized by red-colored eyes, which is mediated by the accumulation of sepiapterin, a yellow fluorescent pteridine derivative [42,43]. Sepiapterin is involved in the tetrahydrobiopterin pathway essential for the breakdown of phenylalanine, suggesting that this molecule is conserved throughout evolution. The regulation of sepiapterin reductase activity could therefore be a mechanism to accumulate sepiapterin to make specific body parts fluorescent. However, this must be demonstrated. Whether α crustacyanin [40], β-carboline or sepiapterin also contribute to the green fluorescent cuticula of the slipper lobster (Fig 8B) and other fluorescent Arthropoda has to be investigated. Because the fluorescence in crustacea and some other species is not intense and only becomes visible with intense excitation, it is difficult to conclude a functional purpose (e.g. Figs 6C, 8B, 8C, 8F, 9A, 9D and 9E).

Fluorescence has also been described in segmented worms. The intertidal worm *Eulalia sp*. (Polychaeta) secretes a blue-green fluorescent mucus [21]. The gossamer worm (*Tomopteri*s *spp*., *a* pelagic annelid) uses fluorescence to enhance bioluminescent light [44]. The mucus of the tubeworm *Chaetopterus variopedatus* contains blue and green fluorescence mediated by riboflavin and related derivatives [45,46]. In addition, blue fluorescence and bioluminescence has also been observed in the fire worm *Odontosyllis phosphorea* [47,48]. We also found fluorescence in bristle worms from the families Eunicidae and Sabellidae with so far unknown composition and function (Fig 5D–5F).

Bioluminescence in some crinoid species is known [49], as well as fluorescent substances derived from crinoids [50]. however, no in situ recordings of fluorescent Crinoidea is known yet. We have found several species in which the fluorescence ranges from yellow/greenish tips of the arms to green/red fluorescent bodies (Fig 7A–7H). The origin of fluorescence and its possible ecological benefit still need to be investigated.

Various species from different phyla particularly Cnidaria, but also Porifera, Mollusca or Arthropoda live in symbiosis with photosynthetic algae and/or bacteria, which reveal chlorophyll mediated red fluorescence [51–53]. For example, green and orange fluorescence is found in the skeletal elements of a few sponge species. The fluorescence originates most likely from associated algae within the sponge skeletal elements [54]. We also found green, yellow, and orange fluorescence in sponges, where the origin and function has not been characterized. Isopods and some molluscs, such as giant clams, also live in symbiosis with red fluorescent, photosynthetic microbes [22].

The fluorescence seen for *Tridacna* is therefore most likely associated to the chlorophyll photosystem II of the algae [50,52] or involves porphyrins, which are responsible for the red fluorescence in the shells of some gastropd species. In snails, fluorescence is often found in the cerata, which originate either from ingested food (green fluorescence) or symbiotic algae (red fluorescence) [20]. It could be possible, that the nudibranch *Facelina rhodopos* deposits ingested fluorescent food in its cerata since this snail feeds on the hydrozoan *Millepora* that has a weak fluorescence. Behavioural experiments with *Goniobranchus splendidus* revealed that predators learn to avoid feeding on the unpalatable snail. The avoidance behaviour of the fish is triggered by the yellow rim of the snail [55]. The red fluorescent rim of *Nembrotha kubaryana* may, therefore, have similar functions. While ingested or attached symbionts explain the red fluorescence of snails and giant clams, the chemical composition of yellow and red fluorescent rims in *Goniobranchus splendidus* and *Nembrotha kubaryana* must be investigated.

Green and red fluorescence is also common in fish [56]. Red fluorescence around 600 nm, for example, has been reported in over 30 reef fish from more than 16 genera and 5 families [57]. The red fluorescence has been associated with guanine crystals, frequently produced in iridophores and has been found in pipefish (Fig 11), triplefins, blennies, and gobies [14,57]. Red fluorescence is found in the iris and parts of the head and thorax and more rarely in fins. It has been shown that the black-faced blenny *Tripterygion delaisi*, which has red fluorescent irises can perceive and respond to red fluorescence [14–16]. Perception of possible fluorescent rivals leads to an increase in aggressive behavior in the red-eyed wrasse *Cirrhilabrus solorensis* [12,13,17]. In crypto-benthic fish, such as the scarlet frogfish *Antennatus coccineus* and the leopard flounder *Bothus pantherinus* (Fig 11) fluorescence may facilitate background matching (camouflage) [8,56]. This has recently been demonstrated in scorpionfish for adjusting red fluorescence to background luminance [58].

We also photographed yellow fluorescent corals that live near the water surface (≤ 15 m (Fig 3). So far, no yellow GFP-like fluorescent protein has been isolated and cloned from stony corals [6]. Also, it was rather known that yellow fluorescent corals can occur in mesophotic reefs [59]. This could be useful for further studies on fluorescent proteins from stony corals. However, yellow fluorescence could also be caused by red and green fluorescent proteins that are colocalized. Isolation and characterisation of the fluorescent proteins from these corals could provide further information.

In summary, this study describes fluorescent marine organisms of different species in which fluorescence has not been published before in scientific literature. A total of 27 species, in which fluorescence has not been described in scientific literature before are added to the list of known fluorescent marine organisms. Three of these species belong to the phylum Porifera, seven to the phylum Mollusca, three to the subphylum Crustacea, three to the phylum of Annelida, two to the class Ascidiacea, and three to the subphylum Vertebrata. We describe the first cases of fluorescence in Octopoda and Ascidiacea, and show fluorescence in Nudibranchia, where the fluorescence–unlike most previous observations—is not linked to ingested food. This study, therefore, extends the palette of fluorescence in marine species. It shows that fluorescence likely is a common phenomenon and that its diversity is not limited to cnidarians. Systematically searching marine biodiversity hotspots with blue or UV lights will, therefore, likely result in the discovery of more fluorescent species and molecules, which will help understand the diverse roles fluorescence may play in marine ecosystems.

## Supporting information

S1 File Example for the digital processing. (DOCX)

S2 File (DOCX)
